# The Effect of Early Life Factors and Early Interventions on Childhood Overweight and Obesity 2016

**DOI:** 10.1155/2017/3642818

**Published:** 2017-02-05

**Authors:** Li Ming Wen, Chris Rissel, Gengsheng He

**Affiliations:** ^1^Department of Endocrinology and Metabolism, Shanghai 10th People's Hospital, University of Tongji, Shanghai 200072, China; ^2^Sydney Medical School, University of Sydney, Sydney, NSW 2006, Australia; ^3^School of Public Health, Fudan University, Shanghai 200032, China; ^4^Health Promotion Service, Sydney Local Health Districts, Sydney, NSW 2050, Australia; ^5^Prevention Research Collaboration, Sydney School of Public Health, University of Sydney, Sydney, NSW 2006, Australia

Increasing prevalence of infant and childhood obesity around the world is a major public health concern [1]. If current trends continue, the number of overweight or obese infants and young children globally will increase to 70 million by 2025 [2]. Without the implementation of secondary prevention or early intervention programs, obese infants and young children will likely continue to be obese during childhood, adolescence, and adulthood. Determining the early life factors associated with obesity is key to developing early intervention strategies and preventing obesity among young children in the first few years of life. Thus, there is an urgent need for a better understanding of the effect of early life factors on overweight and obesity and, more importantly, for the development of effective early interventions.

Over recent years there have been an increasing number of research studies on early life factors and their effects on the childhood obesity. Research into the effects of early interventions on childhood overweight and obesity has also been gathering momentum [3, 4]. In 2015, we edited the first special issue on “The Effect of Early Life Factors and Early Interventions on Childhood Overweight and Obesity” [5] (https://www.hindawi.com/journals/jobe/2015/964540/), which included a number of interesting and important studies. It has become apparent that early infant feeding practices, children's eating habits, and television viewing time are among the most identifiable factors contributing to the early onset of childhood obesity. Providing parents with evidence-based advice and recommendations can improve parental knowledge and practice regarding obesity prevention. However, much more needs to be done to better understand the major contributing factors to obesity in the early years and what interventions can be effective for preventing childhood obesity. With this in mind we selected this theme again for this second special issue.

There was a good response to the call for this special issue, with various research ideas and pilot intervention studies on obesity prevention in the early years. We highly commend the authors for their well written papers exploring a range of issues related to early life factors associated with childhood obesity, which include epidemiological investigations, research reviews, and intervention studies at early life-stages with various populations. In this editorial we wish to highlight some interesting lessons from these studies.

Hughes et al. investigated the influence of feeding styles and food parenting practices on low-income children's weight status over time with 129 Latina parents and their children participating in a longitudinal study. Children were assessed at baseline (4 to 5 years old) and again eighteen months later. At each time point, parents completed questionnaires and height and weight measures were taken on the child. Their results showed that the indulgent feeding style (parent report at baseline) was associated with increased child BMI score eighteen months later compared to other feeding styles. Authoritative, authoritarian, and uninvolved feeding styles were not significantly associated with increased child BMI score. The study was the first to investigate the impact of feeding styles on child weight status over time and highlights the importance of feeding style in childhood obesity prevention and the role of indulgent feeding in predicting later increases in children's weight status. We believe that the interplay between feeding styles and feeding practices in influencing child weight status needs to be further explored.

No or short duration of breastfeeding is also related to feeding practices and has been identified as an important risk factor for childhood obesity [6]. Adams et al. examined breastfeeding practices among Native Hawaiians and Pacific Islanders (NHPI) women through searching seven databases and reference lists based on predetermined criteria and conducted a meta-analysis. They found only nine studies met the inclusion criteria and most studies were cross-sectional with no randomized or quasi-randomized control trials being conducted. Their results indicated that 47% NHPI women initiated breastfeeding with 41% breastfeeding exclusively which were below the recommended national and international goals and guidelines. The study highlighted breastfeeding practices among NHPI women are heterogeneous and critical disparities exist among certain NHPI subgroups and additional research needs to be conducted to determine the reasons for the disparity. Future studies should be conducted to explore barriers and enablers of breastfeeding among the various subpopulations of NHPI women. We believe that the study findings can be applied to other populations, and multicomponent, multilevel strategies are needed to support breastfeeding practices.

Recognising the role of childcare settings in improving obesity-related behaviours, Vinci et al. conducted a feasibility and acceptability study of training teachers to incorporate student intervention curriculum materials in classroom-based activities in order to promote physical activity (PA) in childcare classrooms. They conducted an evidence scan, key informant, and focus group interviews with childcare directors and staff and environmental self-assessment of childcare facilities to inform the design of the training curriculum. Feedback from the interviews indicated that childcare providers believed in the importance of teaching children about PA and were supportive of training teachers to incorporate PA into classroom settings. The Promoting PA in Childcare Setting Curriculum was developed. and training was implemented with 16 teachers. Participants reported a positive experience with the hands-on training and reported acquiring new knowledge that they intended to implement in their childcare settings. Their findings highlight the feasibility of working with childcare staff to develop PA training and curriculum. We believe that there is a great potential for childcare staff to incorporate intervention curriculum materials in classroom-based activities in order to promote physical activity in childcare classrooms.

Acknowledging that parents play a critical role in obesity prevention [7], Foster et al. assessed the feasibility of a parent mentor model of intervention for early childhood obesity using positive deviance-based methods to inform the intervention. In their pilot study parent-child dyads (children aged: 2–5 years) with children whose body mass index (BMI) was ≥95th percentile were randomized to parent mentor intervention or community health worker comparison. The child's height and weight were measured at baseline, after the six-month intervention, and six months after the intervention. Their results showed that, at the end of the intervention, the BMI *z*-score for the parent mentor group was 2.48 (SD = 0.58) and for the community health worker group it was 2.45 (SD = 0.91), both reduced from baseline. We believe that a parent or community health worker mentor program is feasible and both achieved sustained effects on adiposity in an obese child. But further well designed studies with a larger sample size are needed.

Interventions targeting parents and focused on modifiable factors to prevent obesity and promote healthy growth in the first 1000 days of life have received attention in recent years. Utilizing nutrition education theories, Uesugi et al. described the design of a digital-based nutrition guidance system targeted to first-time mothers to prevent obesity during the first two years. The multicomponent system consists of scientifically substantiated content, tools, and telephone-based professional support delivered in an anticipatory and sequential manner via the internet, email, and text messages, focusing on educational modules addressing the modifiable factors associated with childhood obesity. Digital delivery formats leverage consumer media trends and provides the opportunity for scale-up, unavailable to previous interventions reliant on resource heavy clinic and home-based counselling. Designed initially for use in the United States, this system's core features are applicable to all contexts and constitute an approach fostering healthy growth, not just obesity prevention. The multicomponent features, combined with a global concern for optimal growth and positive trends in mobile internet use, represent this system's future potential to affect change in nutrition practice in developing countries. We believe that scale-up of interventions to global populations is necessary to reverse trends in weight status among infants and toddlers, and large scale dissemination will require understanding of effective strategies.

In conclusion, the early onset of childhood overweight and obesity requires the implementation of secondary prevention or early intervention programs to commence as early as possible. A better understanding of the early life factors associated with childhood obesity can help inform the development of appropriate intervention programs. We recommend the original papers in this second special issue addressing “The Effect of Early Life Factors and Early Interventions on Childhood Overweight and Obesity.” We also recognize that much more research is needed to address this substantial public health problem.

*Li Ming Wen* *Chris Rissel* *Gengsheng He*
